# Natriuretic peptide receptor‐C‐mediated attenuation of vascular smooth muscle cell hypertrophy involves Gqα/PLCβ1 proteins and ROS‐associated signaling

**DOI:** 10.1002/prp2.375

**Published:** 2017-12-18

**Authors:** Ashish Jain, Madhu B. Anand‐Srivastava

**Affiliations:** ^1^ Department of Pharmacology and Physiology Faculty of Medicine University of Montreal Québec Canada

**Keywords:** Gqα/PLCβ proteins, hypertrophy, NPR‐C, SHR, VSMC

## Abstract

Hypertension is associated with vascular remodeling due to hyperproliferation and hypertrophy of vascular smooth muscle cells (VSMC). Recently, we showed the implication of enhanced expression of Gqα and PLCβ1 proteins in hypertrophy of VSMCs from 16‐week‐old spontaneously hypertensive rats (SHR). The aim of this study was to investigate whether C‐ANP_4‐23_, a natriuretic peptide receptor‐C (NPR‐C) ligand that was shown to inhibit vasoactive peptide‐induced enhanced protein synthesis in A10 VSMC could also attenuate hypertrophy of VSMC isolated from rat model of cardiac hypertrophy and to further explore the possible involvement of Gqα/PLCβ1 proteins and ROS‐mediated signaling in this effect. The protein synthesis and cell volume, markers of hypertrophy were significantly enhanced in VSMC from 16‐week‐old SHR compared with age‐matched WKY rats and C‐ANP_4‐23_ treatment attenuated both to WKY levels. In addition, C‐ANP_4‐23_ treatment also attenuated the enhanced expression of AT1 receptor, Gqα, PLCβ1, Nox4, and p47^phox^ proteins, the enhanced activation of EGFR, PDGFR, IGF‐1R, enhanced phosphorylation of ERK1/2/AKT and c‐Src in VSMC from SHR. Furthermore, the enhanced levels of superoxide anion and NADPH oxidase activity exhibited by VSMC from SHR were also attenuated to control levels by C‐ANP_4‐23_ treatment. These results indicate that C‐ANP_4‐23_ via the activation of NPR‐C attenuates VSMC hypertrophy through decreasing the overexpression of Gqα/PLCβ1 proteins, enhanced oxidative stress, increased activation of growth factor receptors, and enhanced phosphorylation of MAPK/AKT signaling pathways. Thus, it can be suggested that C‐ANP_4‐23_ may be used as a therapeutic agent for the treatment of vascular complications associated with hypertension and atherosclerosis.

**Abbreviations**
ANP: atrial natriuretic peptide
BNP: brain natriuretic peptide
C‐ANP_4‐23_: a natriuretic peptide receptor‐C (NPR‐C) agonist
CNP: C‐type natriuretic peptide
EGFR: epidermal growth factor receptor
GPCR: G‐protein‐coupled receptor
IGFR: insulin‐like growth factor receptor
NPR‐C: natriuretic peptide receptor‐C
PDGFR: platelet‐derived growth factor receptors
PKC: protein kinase C
PLC‐β: phospholipase C‐β
SHR: spontaneously hypertensive rats
VSMC: vascular smooth muscle cells
WKY rats: Wistar Kyoto rats

## 1 INTRODUCTION

Hypertrophy and proliferation of vascular smooth muscle cells have been shown as important contributors of vascular remodeling and are important hallmarks of vascular disease such as atherosclerosis, restenosis, and hypertension. Angiotensin II (Ang II) is one of the pathophysiological factors that promote VSMC hypertrophy through the activation of several signaling pathways including MAP kinase, PI3Kinase, phosphatidyl inositide, and tyrosine kinase.^1^ Ang II through the interaction with AT1 receptor activates, phospholipase C‐β (PLC‐β) that catalyzes the formation of 2‐second messengers inositol 1,4,5‐trisphosphate [Ins(1,4,5)P3] (IP_3_) and diacylglycerol (DAG) from inositol 1,4,5‐trisphosphate [Ins(1,4,5)P3] (IP_3_ 2) and results in the activation of protein kinase C (PKC).^3^, ^4^ The Gqα and associated signaling has been shown to contribute to Ang II‐induced VSMC hypertrophy.^5^ We recently showed that VSMC from 16‐week‐old spontaneously hypertensive rats (SHR) exhibit enhanced expression of Gqα, PLCβ1, and PKCδ proteins that contribute to VSMC hypertrophy.^6^, ^7^, ^8^

Natriuretic peptides (NPs) comprise a family of three peptide hormones; atrial natriuretic peptide (ANP), brain natriuretic peptide (BNP), and C‐type natriuretic peptide (CNP)^9^, ^10^ and regulate a variety of physiological functions including blood pressure through their interaction with natriuretic peptide receptors (NPRs). Three subtypes of NPRs have been identified: NPR‐A,^11^ NPR‐B,^12^, ^13^.and NPR‐C.^14^ NPR‐A and NPR‐B are membrane guanylyl cyclase receptors, whereas NPR‐C does not possess guanylyl cyclase activity and is coupled to adenylyl cyclase inhibition through the inhibitory guanine nucleotide regulatory protein Gi,^14^, ^15^ or to activation of phospholipase C (PLC).^16^

ANP has been shown to act as an autocrine/paracrine modulator of cardiac hypertrophy and remodelling.^17^, ^18^, ^19^ We have earlier demonstrated that C‐ANP_4‐23,_ an agonist that interacts specifically with NPR‐C and small peptide fragments of cytoplasmic domain of NPR‐C with Gi activator sequences inhibited vasoactive peptide‐induced hypertrophy of A10 VSMC.^20^ However, whether C‐ANP_4‐23_ could also attenuate hypertrophy of VSMC from SHR, a rat model that exhibits cardiac hypertrophy remains obscure. This study therefore investigates the effect of C‐ANP_4‐23_ on the hypertrophy of VSMC from SHR and to explore the implication of different signaling molecules including oxidative stress, c‐Src, growth factor receptors, MAP kinase/PI3kinase, and Gqα/PLCβ1 proteins in mediating this effect.

## 2 MATERIALS AND METHODS

### 2.1 Materials

A ring‐deleted analog of ANP; C‐ANP_4‐23_ was purchased from Bachem (Torrance, CA). Leucine, L‐(4,5‐3H(N)) was purchased from Perkin Elmer (Boston, MA). Polyclonal AT‐1 (N‐10), Monoclonal Gqα antibody (10), monoclonal PLC‐β1 antibody (D‐8), monoclonal (phosphor)‐ERK1/2 (phospho‐specific‐tyrosine204) antibody, polyclonal ERK1/2 antibody (C‐14), monoclonal dynein IC1/2 antibody (74‐1), Polyclonal EGFR, IGF‐1R (phospho)‐c‐Src (phospho‐specific‐tyrosine‐419), PDGFR and (phospho)‐IGF‐1R (phospho‐specific‐tyrosine1165/1166) antibodies, and Western blotting reagents were purchased from St Cruz Biotech (Santa Cruz, CA, USA). Polyclonal (phospho)‐EGFR antibody (phospho‐specific‐tyrosine‐1173), polyclonal (phospho)‐PDGFR (phospho‐specific‐tyrosine 857) were purchased from Cell Signaling Technology (Danvers, MA, USA). Gq inhibitor (GqI), YM‐254890 was purchased from Wako Inc (Osaca, Japan).

### 2.2 Cell culture and incubation

Aortic VSMCs from 16‐week‐old SHR and age‐matched WKY rats were cultured as described previously^21^ and contained high levels of smooth‐muscle‐specific actin.^7^, ^21^, ^22^ The cells after incubation at 37°C in 95% air and 5% CO_2_ humidified atmosphere in Dulbecco's modified Eagle's medium (DMEM) (with glucose, l‐glutamine, and sodium bicarbonate) containing 1% antibiotics (containing penicillin, streptomycin, and amphoterecin B) and 10% heat‐inactivated fetal bovine serum (FBS) were passaged upon reaching confluence with 0.5% trypsin containing 0.2% EDTA and utilized between passages 2 and 8. Confluent cells from SHR and WKY rats after starving for 24 hours in DMEM without FBS at 37°C were further incubated for 24 hours in the absence or presence of 0.1 μmol·L^−1^ C‐ANP_4‐23_. After incubation, the cells were washed twice with ice‐cold phosphate‐buffered saline (PBS) and lysed in a 200 μL buffer containing 25 mmol·L^−1^ Tris‐HCL (pH 7.5), 25 mmol·L^−1^ NaCl, 1 mmol·L^−1^ sodium orthovanadate, 10 mmol·L^−1^ sodium fluoride, 10 mmol·L^−1^ sodium pyrophosphate, 2 mmol·L^−1^ EDTA, 1 mmol·L^−1^ phenylmethylsulfonyl fluoride, 10 μg/mL aprotinin, 1% Triton X‐100, 0.1% sodium dodecyl sulfate, and 0.5 μg/mL leupeptin on ice as described earlier.^23^ The cell lysates were centrifuged at 12,000*g* for 15 minutes at 4°C, and the supernatants were used for Western blot analysis. Cell viability was checked by the trypan blue exclusion technique and indicated that >90%–95% cells were viable. All animal procedures used in this study were approved by the Comite de Deontologie de L'Experimentation sur les Animeaux (CDEA) of the University of Montreal (#99050). The investigation conforms to the Guide for the Care and Use of Laboratory Animals published by the US National Institutes of Health (Guide, NRC 2011).

### 2.3 Western blotting

The levels of protein expression and phosphorylation were determined by Western blotting as described previously.^6^, ^23^ After SDS‐PAGE, the proteins were transferred to a nitrocellulose membranes and the blots were washed with PBS containing 0.1% Tween‐20 (PBST) and then incubated with respective primary antibodies against AT1(sc‐1173), Gqα(sc‐393), PLCβ1 (sc‐205), Nox4 (sc‐21860), p47phox (sc‐17845), p‐c‐Src (sc‐16846), c‐Src (s‐18), PDGFR (sc‐432), EGFR (sc‐03), p‐IGFR(sc‐101704), IGFR(sc‐713), p‐PDGFR (Y849), p‐EGFR (Y1006) using different dilutions ranging from 1:500 to 1:2000 at 4°C for overnight. The blots were washed and incubated with horseradish peroxidase conjugated goat anti‐rabbit IgG secondary antibody for 1 hour at room temperature. The blots were then washed with PBS and proteins were detected using enhanced chemiluminescence (ECL) Western blotting detection reagents (Santa Cruz, CA, USA). Blots were reprobed with Dynein (sc‐13524) as loading controls. Quantitative analysis of the proteins was performed by densitometric scanning of the autoradiographs as described earlier^24^ using the enhanced laser densitometer (LKB Ultroscan XL, Pharmacia, Dorval, Qc, Canada) and quantified using the gel‐scan XL evaluation software (version 2.1) from Pharmacia.

### 2.4 Methyl‐[^3^H] leucine incorporation

Protein synthesis was determined by the incorporation of [^3^H] leucine into the cells as described earlier.^6^, ^7^, ^20^ Subconfluent VSMC from SHR and WKY were serum deprived for 24 hours and were then incubated in the absence or presence of 0.1 μmol·L^−1^ C‐ANP_4‐23_ and [^3^H]leucine (2 μCi per well) for another 24 hours. After the incubation, the cells were harvested and radioactivity was determined by liquid scintillation counter.

### 2.5 Cell volume measurement

Cell volume measurement was performed as described earlier.^7^VSMCs from 16‐week‐old SHR and age‐matched WKY rats were grown to 50% confluence in cell imaging dish (35 × 10 mm). Cells were serum deprived for 24 hours to induce cell quiescence and were incubated for 24 hours in the absence or presence of C‐ANP_4‐23_ (0.1 μmol·L^−1^). The cells were then washed twice and fixed with 10% formalin for 1 hour in 40°C and further incubated for 45 minutes at room temperature with whole cell stains reagent using Thermo Scientific Cellomics Whole Cell Stains (green). The volume of VSMCs was evaluated by three‐dimensional live cell microscopy imaging using Zeiss LSM‐T‐PMT 700 (Zen 2012), Objective Plan‐Apochromat 63x/1.40 Oil DIC, and 40x/1.40 Oil DIC. The three‐dimensional microscopy datasets interpretation was performed with the software Imaris (Bitplane).

### 2.6 Determination of superoxide anion production

Basal superoxide anion production in VSMC was measured using the lucigenin‐enhanced chemiluminescence method with low concentration (5 μmol/L) of lucigenin as previously described.^7^, ^23^, ^25^ VSMC from SHR and WKY rats were incubated in the absence and presence of C‐ANP_4‐23_ (0.1 μmol·L^−1^) for 24 hours. After the treatment, the cells were washed in oxygenated Kreb–Hepes buffer and placed in scintillation vials containing lucigenin solution. The emitted luminescence was measured with a liquid scintillation counter (Wallace 1409: Perkin Elmer Life Science, St. Laurent, Quebec, Canada). for 5 minutes. The average luminescence value was estimated, the background value subtracted and the result was divided by the total protein mass of each sample.

### 2.7 NADPH oxidase activity determination

The activation of NADPH oxidase activity in the samples was assessed by adding 10^−4^ mol/L NADH (Sigma‐Aldrich) to the vials before counting. Luminescence induced by basal O_2_^–^ was then subtracted from the luminescence value induced by NADH.^7^

### 2.8 Statistical analysis

The number of independent experiments is reported. Each experiment was conducted at least 4 times using separate cell population. All data are expressed as the mean ± SD. Comparisons between groups were made with one way analysis of variance (ANOVA) followed by Dunnett tests using GraphPad Prism5 software. Results were considered significant at a value of *P* < .05.

## 3 RESULTS

### 3.1 C‐ANP_4‐23_ attenuates hypertrophy of VSMC from SHR

**Figure 1:**
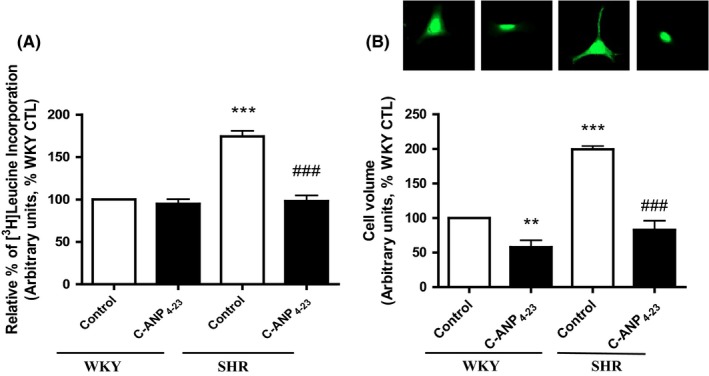
Effect of C‐ ANP_4‐23_ treatment on enhanced protein synthesis and cell volume in VSMC from SHR. Confluent VSMC from 16‐week‐old SHR and age‐matched WKY rats were incubated in the absence or presence of C‐ANP_4‐23_ (0.1 μmol·L^−1^) for 24 hours, Protein synthesis (A) and cell volume (B) were determined as described in “Materials and Methods.” Results are expressed as a % of WKY CTL, taken as 100%. Values are means ± SEM of five separate experiments using different cell populations from different animals. **P < .01, ***P < .001 vs WKY CTL, ^###^ *P* < .001 vs SHR CTL

We earlier showed that C‐ANP_4‐23_ attenuated vasoactive peptide‐induced enhanced protein synthesis in A10 VSMC.^20^ To investigate if C‐ANP_4‐23_ could also attenuate VSMC hypertrophy in animal model of hypertrophy, the effect of C‐ANP_4‐23_ on protein synthesis was examined in VSMC from SHR and WKY rats and the results are shown in Figure 1A. Protein synthesis as determined by leucine incorporation in VSMC from SHR was enhanced by about 80% as compared to WKY rats and C‐ANP_4‐23_ treatment attenuated it to control levels. On the other hand, C‐ANP_4‐23_ treatment did not have any significant effect on protein synthesis in VSMC from WKY rats.

We also determined the effect of C‐ANP_4‐23_ on cell volume, another marker of VSMC hypertrophy and the results are shown in Figure 1B. The cell volume was enhanced by about 100% in VSMC from SHR as compared to WKY, and this enhanced cell volume was attenuated by C‐ANP_4‐23_ treatment by about 80%. In addition, C‐ANP_4‐23_ treatment also decreased basal cell volume in VSMC from WKY rats by about 40%.

### 3.2 C‐ANP_4‐23_ attenuates the enhanced expression of Gαq and PLCβ1 proteins in VSMCs from SHR

**Figure 2:**
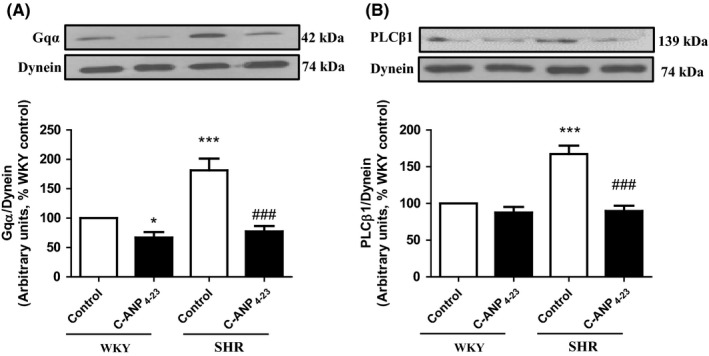
Effect of C‐ANP_4‐23_ treatment on enhanced levels of Gqα and PLCβ1 in VSMC from SHR. Confluent VSMC from 16‐week‐old SHR and age‐matched WKY rats were incubated in the absence or presence of C‐ANP_4‐23_ (0.1 μmol·L^−1^) for 24 hours. The cell lysates were prepared and subjected to Western blotting using specific antibodies against Gqα (A) and PLCβ1 (B) as described in “Materials and Methods.” Dynein was used as a loading control. The protein bands were quantified by densitometric scanning. The results are expressed as a % of WKY, which is taken as 100%. Values are mean ± SEM of five separate experiments using different cell populations from different animals. *P < .05, ***P < .001 vs WKY CTL, ^###^ *P* < .001vs SHR CTL

A role of enhanced expression of Gqα and PLCβ1 proteins in hypertrophy of VSMC from SHR has been recently shown.^6^, ^7^ To investigate if C‐ANP_4‐23_‐induced attenuation of VSMC hypertrophy in SHR is also due to the inhibition of enhanced expression of Gqα and PLCβ1 proteins, we examined the effect of C‐ANP_4‐23_ treatment on the expression of Gqα and PLCβ1 proteins in VSMC from SHR and WKY rats and the results are shown in Figure 2. The expression of Gqα (A) and PLCβ1 (B) was enhanced by about 80% and 75%, respectively, in VSMC from SHR as compared to WKY rats and C‐ANP_4‐23_ treatment almost completely attenuated the enhanced expression to control levels.

**Figure 3:**
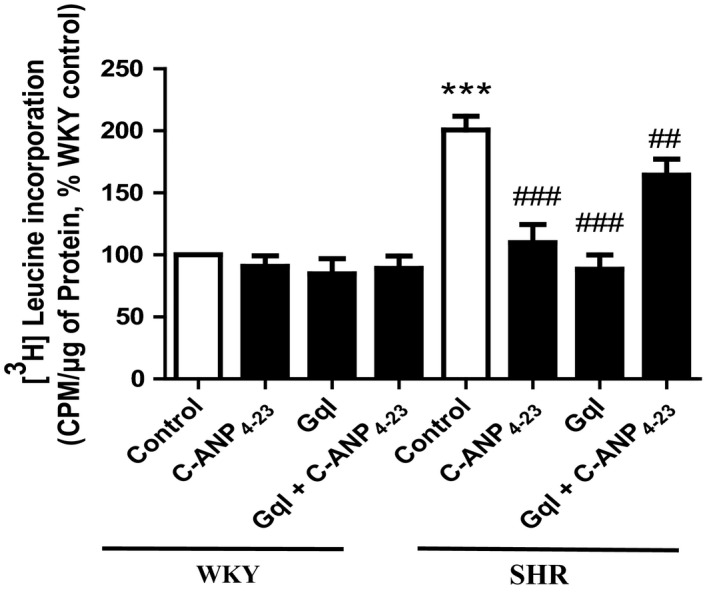
Effect of C‐ANP_4‐23_ and/or GqI treatment on proteins synthesis in VSMCs from SHR and age‐matched WKY rats. VSMC from 16‐week‐old SHR and age‐matched WKY rats were preincubated in the absence (control) or presence of Gq inhibitor YM‐245890 (10 μmol·L^−1^) for 1 hour prior to the treatment with C‐ANP_4‐23_ (0.1 μmol·L^−1^) for 24 hours. Protein synthesis was determined by [^3^H] leucine incorporation as described in “Materials and Methods.” Results are expressed as percentage of control, taken as 100%. Values are means ± SEM of 5 separate experiments using different cell populations from different animals. ***P < .001 vs WKY CTL, ^##^ *P* < .01, ^###^ *P* < .001 vs SHR CTL

To further confirm the requirement of Gqα in C‐ANP_4‐23_‐induced attenuation of hypertrophy of VSMC from SHR, we inhibited Gqα by pretreating the cells with GqI, an inhibitor of Gqα and then examined the effect of inhibition of Gqα on C‐ANP_4‐23_‐induced attenuation of VSMC hypertrophy. Results shown in Figure 3 indicate that GqI as well as C‐ANP_4‐23_ alone attenuated the enhanced protein synthesis in VSMC from SHR to almost control levels, however, when Gqα was inhibited by pretreatment of cells with GqI, the inhibition of enhanced protein synthesis by C‐ANP_4‐23_ was reduced to about 25% suggesting the involvement of Gqα in C‐ANP_4‐23_‐induced attenuation of VSMC hypertrophy.

### 3.3 C‐ANP_4‐23_ attenuates the enhanced expression of AT1 receptor in VSMC from SHR

**Figure 4:**
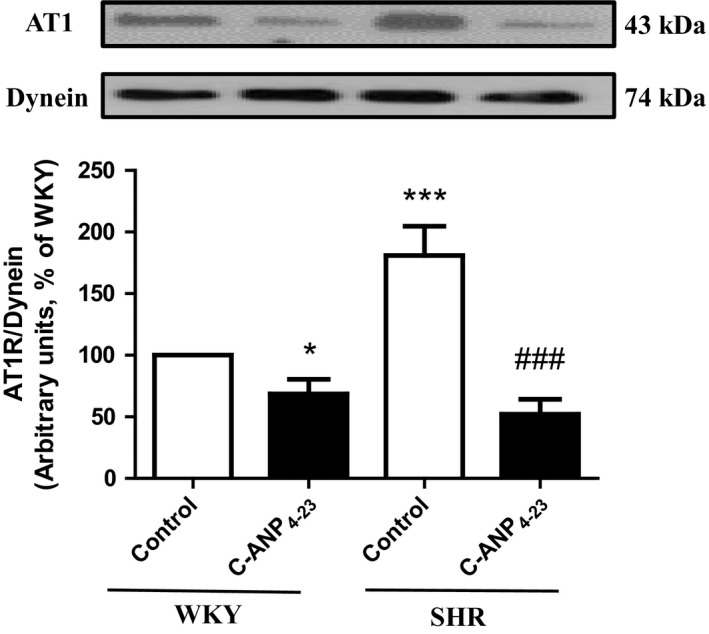
Effect of C‐ANP4‐23 treatment on enhanced expression of AT1 receptor in VSMCs from SHR. Confluent VSMC from 16‐week‐old SHR and age‐matched WKY rats were incubated in the absence or presence of C‐ANP_4‐23_ (0.1 μmol·L^−1^) for 24 hours. The cell lysates were prepared and subjected to Western blotting using specific antibodies against AT1 as described in “Materials and Methods.” Dynein was used as a loading control. The proteins were quantified by densitometric scanning as described in Materials and Methods. Results are expressed as a % of WKY CTL, taken as 100%. Values are means ± SEM of 5 separate experiments using different cell populations from different animals. *P < .05, ***P < .001 vs WKY CTL, ^###^ *P* < .001 vs SHR CTL

Angiotensin II (Ang II) has been shown to induce VSMC hypertrophy.^6^, ^20^ In addition, we recently showed that the enhanced levels of endogenous Ang II through the activation of AT1 receptors contribute to the enhanced expression of Gqα and PLCβ1 proteins as well as VSMC hypertrophy in SHR.^6^ To examine if C‐ANP_4‐23_‐mediated attenuation of VSMC hypertrophy is due to the inhibition of enhanced expression of AT1 receptor, we examined the effect of C‐ANP_4‐23_ treatment on the expression of AT1 receptor in VSMC from SHR and WKY rats. Results shown in Figure 4, indicate that the expression of AT1 receptor was significantly augmented by about 70% in VSMC from SHR as compared to VSMC from WKY rats and this enhanced expression was attenuated to WKY control level by C‐ANP_4‐23_ treatment. In addition C‐ANP_4‐23_ also decreased the expression of AT1 receptor in VSMC from WKY rats by about 30%.

### 3.4 C‐ANP_4‐23_ attenuates enhanced activity of NADPH oxidase and superoxide anion production in VSMC from SHR

**Figure 5:**
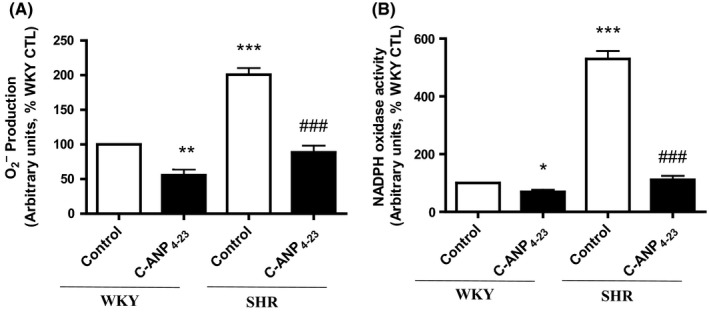
Effect of C‐ANP_4‐23_ treatment on enhanced O_2_^−^ production and NADPH oxidase activity in VSMC from SHR. Confluent VSMC from 16‐week‐old SHR and age‐matched WKY rats were incubated in the absence (control) and presence of C‐ANP_4‐23_ (0.1 μmol·L^−1^) for 24 hours and O_2_^−^ production (A) and NADPH oxidase activity (B) were determined as described in “Materials and Methods”. Results are expressed as % of WKY CTL, taken as 100%. Values are means ± SEM of 5 separate experiments using different cell populations from different animals. *P < .05, **P < .01, ***P < .001 vs WKY CTL, ^###^ *P* < .001 vs SHR CTL

The enhanced oxidative stress has been shown to contribute to VSMC hypertrophy and enhanced expression of Gqα/PLCβ1 proteins in SHR.^7^ To investigate if C‐ANP_4‐23_‐evoked attenuation of VSMC hypertrophy is attributed to its ability to decrease the enhanced oxidative stress, we examined the effect of C‐ANP_4‐23_ on the levels of O_2_^−^ and NADPH oxidase activity in VSMC from SHR and WKY rats. Results shown in Figure 5, demonstrate that the levels of O_2_^−^ (A) and NADPH oxidase activity (B) that were enhanced by approximately 100% and 450%, respectively, in VSMC from SHR as compared to WKY rats were completely attenuated to control WKY levels by C‐ANP_4‐23_ treatment. In addition, C‐ANP_4‐23_ treatment also reduced the levels of O_2_^−^ and NADPH oxidase activity by about 50% and 30%, respectively, in VSMC from WKY rats.

### 3.5 C‐ANP_4‐23_ attenuates the expression of NADPH oxidase subunits in VSMC from SHR

**Figure 6:**
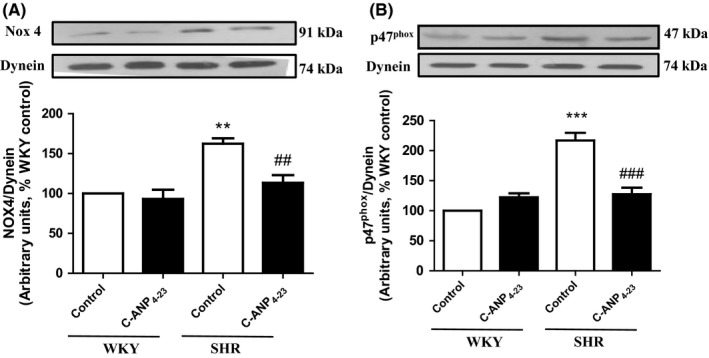
Effect of C‐ANP_4‐23_ treatment on the enhanced levels of NADPH oxidase subunits p47^phox^ and Nox4 in VSMC from SHR. Confluent VSMC from 16‐week‐old SHR and age‐matched WKY rats were incubated in the absence or presence of C‐ANP_4‐23_ (0.1 μmol·L^−1^) for 24 hours. The cell lysates were prepared and subjected to Western blotting using specific antibodies against NOX4 (A) and P47phox (B). Dynein was used as the loading control. The proteins were quantified by densitometric scanning as described in “Materials and Methods.” Results are expressed as % of WKY CTL, taken as 100%. Values are means ± SEM of 5 separate experiments using different cell populations from different animals. **P < .01 ***P < .001 vs WKY CTL, ^##^ *P* < .01, ^###^ *P* < .001 vs SHR CTL

To further explore whether C‐ANP_4‐23_‐induced attenuation of oxidative stress was associated with the decreased expression of the NADPH oxidase subunits, we examined the effect of C‐ANP_4‐23_ treatment on the expression of Nox 4 and p47^phox^ proteins, critical subunits involved in NADPH oxidase activation in VSMC from SHR and WKY rats. Results shown in Figure 6 indicate that the levels of Nox 4 (A) and p47^phox^ (B) that were enhanced by 70% and 120%, respectively, in VSMC from SHR as compared to WKY rats were attenuated to almost control levels by C‐ANP_4‐23_ treatment, whereas the levels of these proteins were not significantly affected in VSMC from WKY rats by this treatment.

### 3.6 C‐ANP_4‐23_ attenuates enhanced activation c‐Src in VSMC from SHR

**Figure 7:**
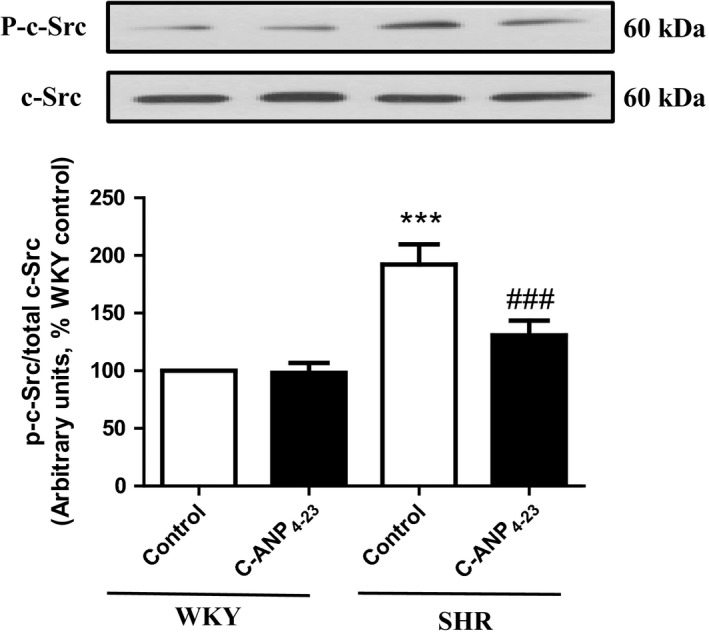
Effect of C‐ANP_4‐23_ treatment on enhanced c‐Src activation in VSMCs from SHR. Confluent VSMC from 16‐week‐old SHR and age‐matched WKY rats were incubated in the absence or presence of C‐ANP_4‐23_ (0.1 μmol·L^−1^) for 24 hours. The cell lysates were prepared and subjected to Western blotting using specific antibodies against (phospho)‐c‐Src (top) and c‐Src (bottom) as described in “Materials and Methods.” The proteins were quantified by densitometric scanning as described in “Materials and Methods.” Results are expressed as a % of WKY CTL, taken as 100%. Values are means ± SEM of 5 separate experiments using different cell populations from different animals. ***P < 0.001 vs WKY CTL, ^###^ *P* < .001 vs SHR CTL

The implication of nonreceptor tyrosine kinase c‐Src in VSMC hypertrophy and augmented expression of Gqα and PLCβ1 proteins in SHR has previously been shown.^7^ To investigate if C‐ANP_4‐23_ mediated antihypertrophic effect is due to the inhibition of the enhanced activity of c‐Src, the effect of C‐ANP_4‐23_ treatment on c‐Src activation was examined in VSMC from SHR and WKY rats and the results are shown in Figure 7. The phosphorylation of Tyr^418^ on c‐Src was increased by almost 70% in VSMC from SHR as compared to WKY rats and C‐ANP_4‐23_ treatment completely attenuated this enhanced phosphorylation to control levels. On the other hand, this treatment did not have any significant effect on c‐Src phosphorylation in VSMC from WKY rats.

### 3.7 C‐ANP_4‐23_ attenuates enhanced phosphorylation of growth factor receptors in VSMCs from SHR

**Figure 8:**
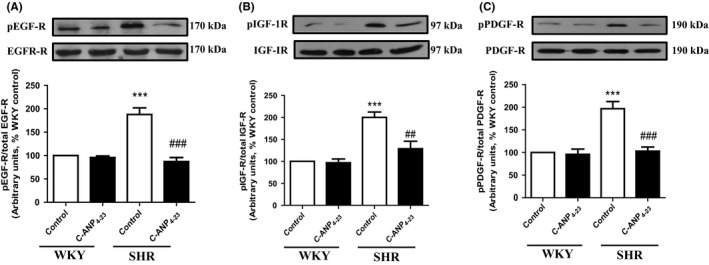
Effect of C‐ ANP_4‐23_ treatment on the hyperphosphorylation of epidermal growth factor receptor (EGFR), insulin‐like growth factor receptor (IGFR), and platelet‐derived growth factor receptor (PDGFR) in VSMC from SHR. Confluent VSMC from 16‐week‐old SHR and age‐matched WKY rats were incubated in the absence or presence of C‐ANP_4‐23_ (0.1 μmol·L^−1^) for 24 hours. The cell lysates were prepared and subjected to Western blotting using specific antibodies against p‐EGFR/EGFR (A), p‐IGF‐1R/IGF‐1R (B), and p‐PDGFR/PDGFR (C). The proteins were quantified by densitometric scanning as described in “Materials and Methods.” Results are expressed as % of WKY CTL, taken as 100%. Values are means ± SD of 5 separate experiments using different cell populations from different animals. ***P < .001 vs WKY CTL, ^##^ *P* < .01, ^###^ *P* < .001 vs SHR CTL

The role of growth factor receptor transactivation in enhanced protein synthesis in SHR has been demonstrated.^7^ Therefore, to explore whether C‐ANP_4‐23_ treatment attenuates VSMC hypertrophy through the inhibition of enhanced activation of growth factor receptors, we examined the effect of C‐ANP_4‐23_ treatment on the phosphorylation of EGFR, IGF‐1R, and PDGFR. Results shown in Figure 8 indicate that the levels of phosphorylated EGFR (A), IGF‐1R (B), and PDGFR (C) were enhanced by 85%, 95%, and 95%, respectively, in VSMC from SHR as compared to WKY rats and this enhanced phosphorylation was attenuated by 90%, 95%, and 90%, respectively, by C‐ANP_4‐23_ treatment. On the other hand, C‐ANP_4‐23_ treatment did not have any significant effect on the phosphorylation of these receptors in WKY rats.

### 3.8 C‐ANP_4‐23_ attenuates enhanced phosphorylation of ERK1/2 and AKT in VSMCs from SHR

**Figure 9:**
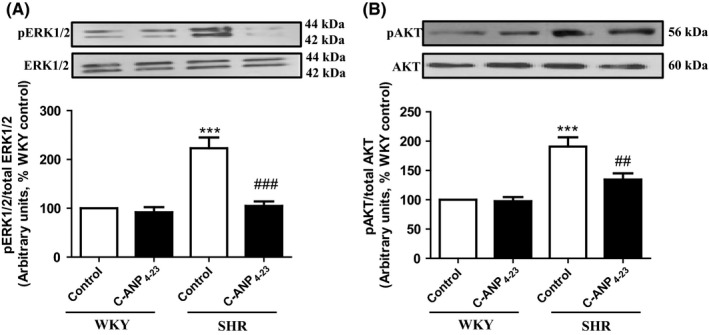
Effect of C‐ANP_4‐23_ treatment on the enhanced phosphorylation of extracellular signal‐regulated kinase ERK1/2 and AKT in VSMC from SHR. Confluent VSMC from 16‐week‐old SHR and age‐matched WKY rats were incubated in the absence or presence of C‐ANP_4‐23_ (0.1 μmol·L^−1^) for 24 hours. The cell lysates were prepared and subjected to Western blotting using specific antibodies against pERK1/2/ERK1/2 (A) and pAKT/AKT (B) as described in “Materials and Methods.” Results are expressed as % of WKY CTL taken as 100%. Values are means ± SEM of 5 separate experiments using different cell populations from different animals. ***P < .001 vs WKY CTL, ^##^ *P* < .01, ^###^ *P* < .001 vs SHR CTL

Since MAP kinase and AKT have been implicated in VSMC hypertrophy from SHR,^6^ it was of interest to investigate if C‐ANP_4‐23_‐evoked attenuation of VSMC hypertrophy is attributed to its ability to inhibit the enhanced activation of ERK1/2 and AKT. To test this, the effect of C‐ANP_4‐23_ treatment on the levels of phosphorylated ERK1/2 (A) and AKT (B) were examined in VSMCs from SHR and WKY rats and the results are shown in Figure 9. The phosphorylation levels of ERK1/2 and AKT that were enhanced by about 120% and 50%, respectively, in VSMC from SHR as compared to WKY rats were completely abolished by C‐ANP_4‐23_ treatment, however, this treatment, did not affect the phosphorylation of ERK1/2 and AKT in VSMCs from WKY rats.

## 4 DISCUSSION

We earlier showed that VSMC from 16‐week‐old SHR exhibit enhanced expression of Gqα and PLCβ1 proteins that contribute to VSMC hypertrophy.^6^, ^7^ We also showed that small peptide fragments of the cytoplasmic domain of NPR‐C and C‐ANP_4‐23,_ an agonist of NPR‐C, attenuated the vasoactive peptide‐induced hypertrophy of A10 VSMC.^20^ However, in this study, we report for the first time that of C‐ANP_4‐23_ treatment attenuates hypertrophy of VSMC from 16‐week‐old SHR, a model of cardiac hypertrophy through the inhibition of enhanced expression of AT1, Gqα/PLCβ1 proteins and ROS and ROS‐mediated c‐Src signaling pathways.

The Gqα protein and associated signaling pathway activated by several hormones such as angiotensin II, endothelin, phenylephrine has also been implicated in the development and progression of cardiac hypertrophy and heart failure.^26^, ^27^, ^28^, ^29^, ^30^ In addition, Gqα and the associated signaling pathways, including the activation of IP3‐Ca^+2^ and DAG‐PKC, have been implicated in the development and progression of VSMC hypertrophy.^5^ We recently showed the role of enhanced expression of Gqα and PLCβ1 in VSMC hypertrophy in SHR.^6^, ^7^ We now show that C‐ANP_4‐23_, an NPR‐C agonist attenuates the enhanced expression of Gqα and PLCβ1 proteins as well as hypertrophy of VSMC from SHR and suggest that C‐ANP_4‐23_‐evoked inhibition of enhanced protein synthesis is attributed to its ability to attenuate the enhanced levels of Gqα and PLCβ1 proteins. The implication of Gqα in C‐ANP_4‐23_‐induced attenuation of VSMC hypertrophy in SHR is further substantiated by our study showing that inhibition of Gqα by a specific inhibitor GqI 31, 32 inhibited the ability of C‐ANP_4‐23_ to completely attenuate the enhanced protein synthesis in these cells. These results are in accordance with the study of Harris et al who have also shown that inhibition of Gqα signaling by GqI reduced VSMC hypertrophy in the aortas of hypertensive rats.^33^ On the other hand, the activation of NPR‐C by C‐ANP_4‐23_ and resultant decreased levels of intracellular cAMP^14^, ^15^ may not be the underlying mechanism contributing to the antihypertrophic effect of C‐ANP_4‐23,_ because the intracellular cAMP levels are shown to be decreased in VSMC from SHR as compared to WKY rats^34^ and therefore may not be responsible for the hypertrophy of these cells because elevating the intracellular levels of cAMP by 8‐Br‐cAMP was shown to attenuate the hypertrophy of VSMC from SHR (unpublished observation).

Furthermore, enhanced levels of endogenous Ang II AT1 and endothelin‐1 ET_A_ were shown to contribute to the enhanced expression of Gqα and PLCβ1 and VSMC hypertrophy in SHR because AT1 and ET_A_ receptor antagonists losartan and BQ123 attenuated the enhanced expression of Gqα, PLCβ1 as well as increased protein synthesis.^6^ In addition, Nakashima et al has also reported the role of Ang II‐induced Gq signaling in vascular hypertrophy.^35^ In this study, we show that C‐ANP_4‐23_ attenuated the enhanced expression of AT1 receptor in VSMC from SHR to control levels and suggest that C‐ANP_4‐23_‐evoked antihypertrophic effect may also be attributed to its ability to decrease the levels of AT1 receptor.

Oxidative stress has been shown to play an integral role in the development of cardiovascular disease, including hypertension.^7^, ^25^, ^36^, ^37^ The implication of ROS in cardiomyocyte and VSMC hypertrophy has been demonstrated in several studies.^38^, ^39^, ^40^ We earlier showed the role of enhanced oxidative stress in the overexpression of Gqα and PLCβ1 proteins in VSMC from SHR.^7^ Our results showing that C‐ANP_4‐23_ treatment of VSMC from SHR attenuated the enhanced levels of O_2_^−^ production, NADPH oxidase activity as well as the increased levels of NADPH oxidase subunits p47^phox^ and Nox4 are consistent with our earlier study showing that in vivo treatment of SHR with C‐ANP_4‐23_ attenuated the enhanced levels of O_2_^−^, NADPH oxidase activity, and the enhanced expression of Nox4, p47phox in aorta, heart as well as in kidney^41^ and suggest that C‐ANP_4‐23_‐induced inhibition of oxidative stress may also play a role in the antihypertrophic effect of C‐ANP_4‐23_.

The role of growth factor receptors in VSMC hypertrophy has been demonstrated by several studies.^42^, ^43^, ^44^ We earlier showed the implication of growth factor receptor activation in enhanced expression of Gqα and PLCβ1 proteins and VSMC hypertrophy in SHR.^22^, ^36^, ^45^ In this study, we demonstrate for the first time that treatment of VSMC from SHR with C‐ANP_4‐23_ attenuated the enhanced phosphorylation of EGFR, PDGFR, and IGF‐1R and suggest that the antihypertrophic effect of C‐ANP_4‐23_ may also be attributed to its ability to attenuate the enhanced activation of growth factor receptors.

We earlier showed the role of c‐Src in the increased expression of Gqα and PLCβ1 proteins and enhanced protein synthesis in VSMC from SHR.^7^ The implication of c‐Src in high glucose‐induced overexpression of Gqα and PLCβ1 in A10 VSMCs has also been reported.^46^ Furthermore, c‐Src has also been shown as the intervening molecule between oxidative stress and growth factor receptor transactivation because *N*‐acetylcysteine, a scavenger of O_2_^−^ inhibited the enhanced phosphorylation of c‐Src,^7^ and c‐Src inhibitor PP_2_, inhibited the enhanced phosphorylation of PDGFR and IGFR in VSMC from SHR.^7^ In this study, we showed that C‐ANP_4‐23_ also attenuated the enhanced activation/phosphorylation of c‐Src to control levels and suggest that C‐ANP_4‐23_‐induced inhibition of c‐Src activation contributes to the attenuation of downstream signaling molecules resulting in the attenuation of hypertrophy of VSMC from SHR.

Earlier studies have shown the implication of MAPK signaling in Gqα‐induced cardiac hypertrophy.^47^ In addition, the role of MAP kinase signaling in enhanced expression of Gqα and PLCβ1 proteins and VSMC hypertrophy induced by vasoactive peptides^20^ and in SHR^6^ is also well documented. In this study, we demonstrate that C‐ANP_4‐23_ treatment of VSMC from SHR attenuates the enhanced phosphorylation of ERK1/2 as well as of AKT and suggest that the antihypertrophic effect of C‐ANP_4‐23_ may be mediated through the inhibition of the enhanced activity of the MAP kinase and PI3K signaling pathway. These results are in concordance with a previous study demonstrating that C‐ANP_4‐23_ and small fragments of the cytoplasmic domain of NPR‐C attenuated vasoactive peptide‐induced hypertrophy of A10 VSMC via MAPK signaling pathway.^20^

**Figure 10:**
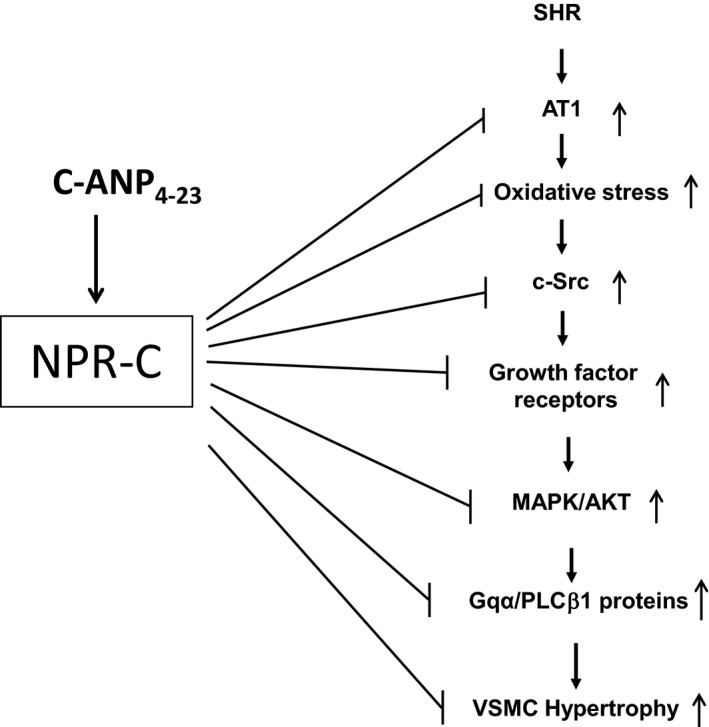
Schematic diagram summarizing the possible signaling mechanisms by which C‐ANP_4‐23_ attenuates the hypertrophy of VSMC from SHR

In conclusion, this study shows for the first time that C‐ANP_4‐23_ through the activation of NPR‐C‐ attenuates overexpression of AT1 receptor and all the signaling molecules including oxidative stress, c‐Src and growth factor receptor activation as well as MAPK/AKT that were shown to be implicated in the enhanced expression of Gqα and PLCβ1 proteins in VSMC from SHR and VSMC hypertrophy.^6^, ^7^ Thus, it may be suggested that C‐ANP_4‐23_‐induced attenuation of the increased expression of Gqα and PLCβ1 proteins and hypertrophy of VSMC from SHR may be attributed to its ability to inhibit the enhanced expression of AT1 receptor, enhanced oxidative stress and downstream signaling pathways (Figure 10) and that C‐ANP_4‐23_ may have protective effect against oxidative stress‐induced vascular complications of hypertension and could be used as a potential therapeutic agent in the treatment of vascular complications associated with hypertension and other cardiovascular diseases.

## DISCLOSURES

No conflicts of interest.

## AUTHOR CONTRIBUTIONS

Jain and Anand‐Srivastava participated in research design, performed data analysis, wrote or contributed to the manuscript. Jain also conducted experiments for the manuscript.
